# A case of transformation of Waldenström’s Macroglobulinemia to diffuse large B cell lymphoma

**DOI:** 10.1093/omcr/omaf290

**Published:** 2026-01-25

**Authors:** Meher B Ali, Kathryn Kline

**Affiliations:** Internal Medicine, University of Maryland Medical Center, 22 S Greene St, Baltimore, Maryland, 21201, United States; Hematology-Oncology, University of Maryland Greenebaum Comprehensive Cancer Center, 22 S Greene St, Baltimore, Maryland, 21201, United States

**Keywords:** case report, lymphoma, large cell lymphoma, waldenström’s macroglobulinemia

## Abstract

Waldenström’s Macroglobulinemia (WM) is a lymphoplasmacytic lymphoma which can rarely transform into diffuse large B cell lymphoma (DLBCL). Usually, the diagnosis of WM precedes DLBCL. A 79-year-old male presented to the hospital for worsening dysphagia and shortness of breath due to a rapidly growing base of tongue mass detected by CT scan. The mass was thought initially to be isolated DLBCL, though incidentally, markedly elevated IgM titers were noted. Biopsy of bone marrow and mass were consistent with histological transformation of WM to DLBCL. Response to treatment was assessed by the size of tongue mass on interval CT scans and IgM levels, both of which responded rapidly to chemotherapy. However, the patient developed early CNS relapse, consistent with the overall poor prognosis of transformed WM.

## Introduction

Waldenström’s Macroglobulinemia (WM) is a low-grade B cell lymphoplasmacytic lymphoma with increase in production of IgM monoclonal protein [1]. IgM monoclonal gammopathy is usually diagnosed with an M spike on serum protein electrophoresis and immunofixation and elevated IgM levels. Bone marrow biopsy establishes a lymphoplasmacytic lymphoma [2]. WM is usually indolent and limited to the bone marrow. Symptoms may occur due to the infiltration of bone marrow and other sites, causing cytopenias, lymphadenopathy, or hepatosplenomegaly. Overproduction of the large monoclonal IgM proteins may also cause hyperviscosity symptoms like headache, thrombosis, or visual impairments [2]. In less than 10% of patients WM transforms into diffuse large B cell lymphoma (DLBCL), which is associated with a poor prognosis [3]. Usually, the diagnosis of WM precedes DLBCL, but here we discuss a case of a patient who was diagnosed concurrently with WM and transformation to DLBCL.

## Case

**Figure 1:**
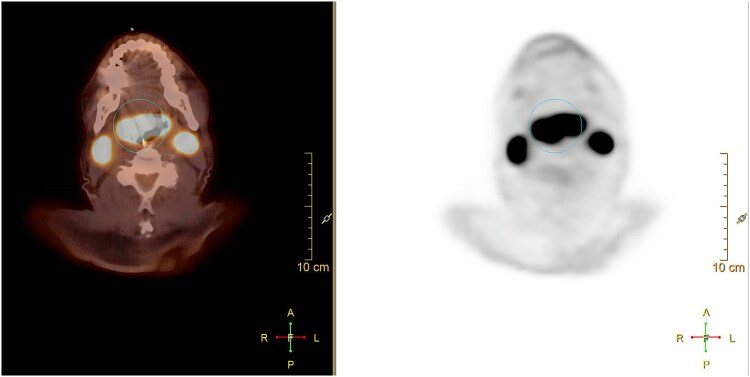
PET/CT showing hypermetabolic base of tongue mass with FDG avid bilateral level IIa cervical lymph nodes.

A 79-year-old male presented to the hospital for worsening dysphagia and shortness of breath for the past one month due to a rapidly growing base of tongue mass detected initially by a CT scan. FDG PET scan showed a right sided mass in the tongue base with increased metabolic activity, with max SUV 25, bilateral large level IIa nodes, with the right one measuring 3.4 ×2.3 cm, SUV of 28, and the left one measuring 2.7 × 2.3 cm, SUV of 33, a subcentimeter right level IIb node with SUV of 12, and a subcentimeter left level III node with SUV of 5 (Fig. 1). There was no evidence of distant metastasis. Biopsy of the tongue mass revealed lambda-restricted diffuse large B cell lymphoma (DLBCL), non-germinal center B cell sub-type (non-GCB). The cells expressed IgM and lambda light chain. Tumor cells were strongly and diffusely positive for CD45, CD20, PAX5, and CD79a, BCL2, and MUM1 and negative for CD3, CD5, and CD10. The Ki67 proliferation index was greater than 90%. FISH studies were negative for BCL2, BCL6, and MYC mutations. MYD88 L265P mutation was detected.

He underwent tracheostomy and gastrostomy tube placement for management of symptoms. During the admission he developed acute kidney injury and was noted to have an elevated globulin gap and M spike on SPEP. IgM was increased to 7900. Laboratory values were hemoglobin 9.9, platelets 335, white blood count 8.5, albumin 4.2, total protein 12.6 (elevated), beta globulin fraction 0.8, lambda light chains 246 (elevated), kappa light chains 24 (elevated), kappa to lambda ratio 0.1 (decreased), and LDH 219. Bone marrow biopsy showed lambda-restricted CD5 and CD10 negative mature B-cell cells and increased lambda-restricted plasma cells, concerning for lymphoplasmacytic lymphoma. FISH studies were positive for 13q and TP53 deletion in 20% of CD138 selected marrow plasma cells. Taken together, findings were concerning for large cell transformation (in the base of tongue) of low-grade B-cell lymphoma with plasmacytic differentiation (in the marrow), consistent with Waldenström’s Macroglobulinemia (WM). The large cell lymphoma did not seem to involve the bone marrow and was staged IE DLBCL (limited to cervical lymph nodes with extension to base of tongue).

We planned him for 6 cycles of rituximab, cyclophosphamide, doxorubicin, vincristine, and prednisone (R-CHOP). ECOG scale was 1 at the beginning of therapy. His NCCN International Prognostic Index (IPI) was 1 (only risk factor of age > 60), which classified him as having low risk disease and modified Staging System for WM (MSS-WM) score was 2, classifying him as intermediate risk. We administered plasmapheresis before starting chemotherapy. We omitted rituximab from cycle 1 due to the risk of worsening hyperviscosity. Due to complications with urosepsis and cholecystitis, we reduced the dosages of doxorubicin and vincristine after 2 cycles and gave him R-mini-CHOP for rest of the cycles. CT scan after cycle 2 showed improving lymphadenopathy. He intermittently received repeated blood transfusions due to persistent anemia and worsening hyperviscosity. ECOG after completion of 6 cycles was 2. We did not pursue CNS prophylaxis due to worsening functionality and limited data on efficacy in preventing CNS relapse.

**Figure 2:**
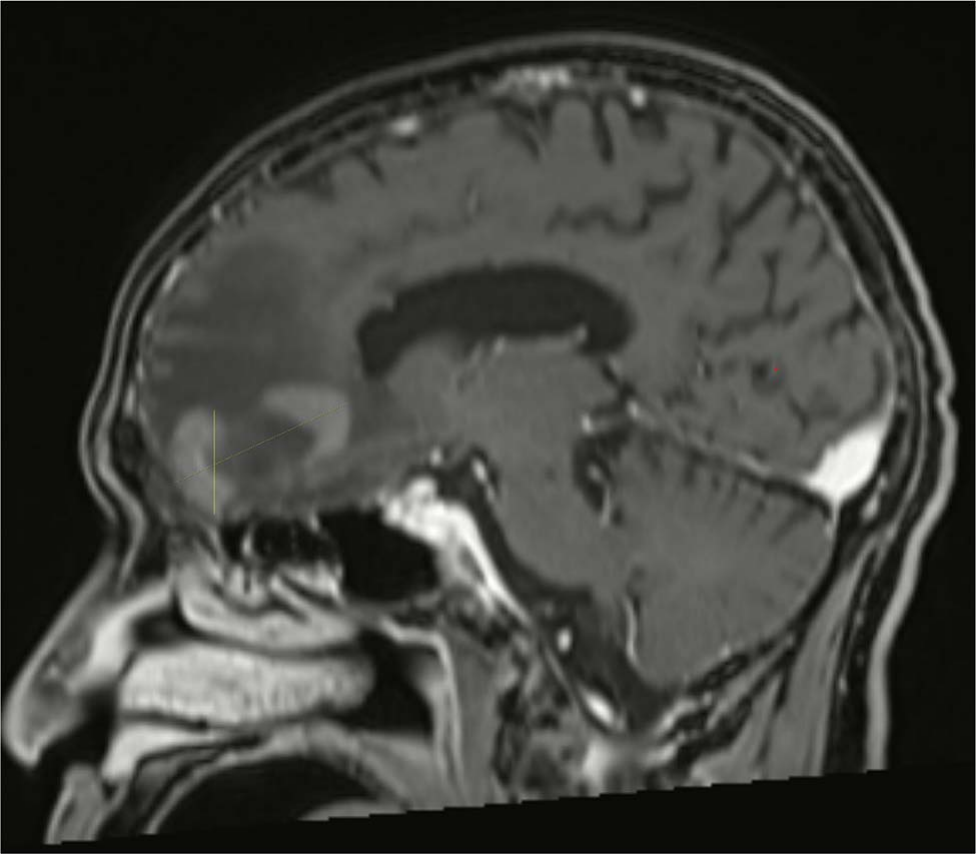
MRI brain showing right frontal lobe mass.

**Figure 3:**
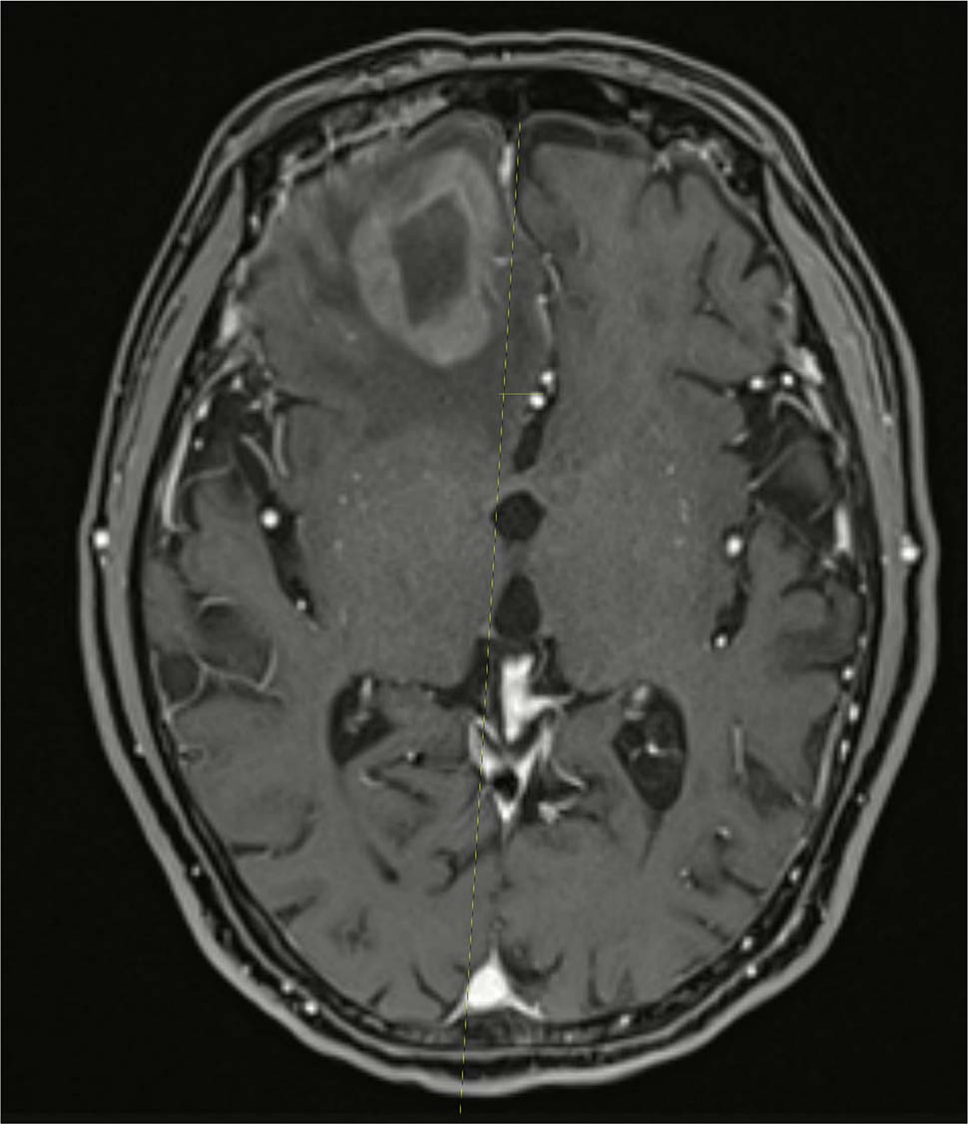
MRI brain showing the right frontal lobe mass causing midline shift.

Almost one month after receiving his sixth cycle of reduced-dose R-CHOP, he was admitted to the hospital due to altered mental status and underwent a brain MRI which showed a heterogeneously enhancing right frontal lobe mass with vasogenic edema and midline shift (Figs 2 and 3). PET scan showed interval resolution of the previously seen tongue base mass and nodes, but metabolically active frontal lobe mass consistent with metastasis to brain from his DLBCL. He was started on corticosteroids and underwent outpatient whole brain radiation therapy treatment, delivering a total of 3000 cGy over 10 fractions. However, he was not considered a good candidate for further chemotherapy targeted at his brain metastases. He and his family elected to switch to palliative and supportive care thereafter and the patient passed away.

## Discussion

Histological transformation (HT) of WM may be suspected when there are rising LDH levels, increasing extranodal involvement, a decrease in IgM level, or advanced IPI score [2]. LPL without MYD88 mutation has a higher occurrence of HT [4, 5]. Interestingly, despite normal LDH levels, extremely high IgM levels, low IPI score, and MYD88 mutation, our patient had HT which makes his case highly unusual. When present, MYD88 mutation has a worse prognosis [5]. Other factors associated with a shorter overall survival include elevated LDH, prior receipt of two or more lines of treatment for WM, and HT after 5 years from diagnosis of WM [6].

There have only been a few cases reported in which LPL and transformed DLBCL were diagnosed at the same time. HT is clear when molecular analysis shows similar clonality. In some prior cases the two lymphomas arose from separate clones, indicating that both were present in the same patient independently, a rare possibility which we cannot completely exclude [7, 8, 9]. In our patient, the presence of lambda restricted cells in both the bone marrow and base of tongue mass was consistent with a clonal relationship between the two. PET scan may be helpful in some forms of HT such as Ritcher transformation, but it can be associated with false positives, so ultimate diagnosis requires a biopsy [10].

R-CHOP (rituximab, cyclophosphamide, doxorubicin, vincristine, prednisone) is the most common regimen used to treat HT [2]. Despite treatment, prognosis is still poor. Relapse into the CNS occurs rarely with DLBCL but there is a higher occurrence in those with HT. Risk is increased in those with MYD88 mutations, kidney or adrenal involvement, and increased LDH [2, 11, 12]. There is poor CNS penetration of rituximab due to its large size and inability to cross the blood brain barrier. The other drugs in the R-CHOP regimen also have limited CNS penetration [4]. There aren’t clear guidelines on CNS prophylaxis with prior literature showing that intra-thecal chemotherapy may not have benefit. [13, 14] If prophylaxis is considered, intra-thecal chemotherapy may be delayed until primary treatment is completed to avoid delays from potential toxicities [4].

Most of the data in literature regarding HT is from retrospective studies and case reports. It is difficult to do trials or prospective studies due to the rarity of HT.

## Conclusion

WM is an indolent neoplasm, which if asymptomatic, is usually monitored over time without treatment. Our patient presented with a rapidly enlarging base of tongue mass. The mass was thought initially to be isolated DLBCL, but incidentally very high IgM titers were noted, and biopsy of bone marrow and mass were consistent with HT of WM to DLBCL. His tongue mass and IgM levels responded rapidly to CHOP-based chemotherapy but unfortunately he developed early CNS relapse, consistent with the overall poor prognosis of transformed WM. Transformed WM should be considered in patients who have elevated total protein, acute kidney injury, or symptoms of hyperviscosity at the time of DLBCL diagnosis, as patients with transformed WM may need to undergo plasmapheresis or have rituximab omitted during their first treatment cycle in order to avoid worsening hyperviscosity.
